# Retraction: Silicon fertilization counteracts salinity-induced damages associated with changes in physio-biochemical modulations in spinach

**DOI:** 10.1371/journal.pone.0274205

**Published:** 2022-09-14

**Authors:** The PLOS ONE Editors

The *PLOS ONE* Editors retract this article [1] because it was identified as one of a series of submissions for which we have concerns about authorship, competing interests, and peer review. We regret that the issues were not addressed prior to the article’s publication.

YC, MHS, and HMA did not agree with the retraction. RN, QuZ, SN, NK, KA, AAAH, AA, FK, KS, and QK either did not respond directly or could not be reached.
